# The neonatal gut microbiome and global health

**DOI:** 10.1080/19490976.2024.2352175

**Published:** 2024-05-14

**Authors:** Pinaki Panigrahi

**Affiliations:** Department of Pediatrics, Division of Neonatal Perinatal Medicine, International Microbiome Research, Georgetown University Medical Center, Georgetown, WA, USA

**Keywords:** Gut microbiota, neonatal sepsis, stunting, gut dysfunction, gut permeability, antimicrobial resistance, lactobacillus, prebiotics, synbiotics, probiotics

## Abstract

The role of gut microbiome in health, a century-old concept, has been on the center stage of medical research recently. While different body sites, disease conditions, and populations have been targeted, neonatal and early infancy appear to be the most suitable period for such interventions. It is intriguing to note that, unlike traditional use in diarrhea and maintenance of gastrointestinal health, microbiome-mediating therapies have now addressed the most serious medical conditions in young infants such as necrotizing enterocolitis and neonatal sepsis. Unfortunately, almost all new endeavors in this space have been carried out in the Western world leaving behind millions of neonates that can benefit from such manipulations while serving as a large resource for further learning. In this review, an attempt has been made to quantify the global burden of neonatal morbidity and mortality, examples presented on interventions that have failed as a result of drawing from studies conducted in the West, and a case made for manipulating the neonatal gut microbiome to address the biggest killers in early life. A brief comparative analysis has been made to demonstrate the differences in the gut microbiota of North and South and a large clinical trial of synbiotics conducted by our group in a South Asian setting has been presented. Although challenging, the value of conducting such global health research is introduced with an intent to invite medical scientists to engage in well-planned, scientifically robust research endeavors. This can bring about innovation while saving and serving the most vulnerable citizens now and protecting them from the negative health consequences in the later part of their lives, ultimately shaping a resilient and equitable world as pledged by 193 United Nations member countries in 2015.

## Introduction

In the current special issue, a wide spectrum of topics starting from basic concepts of diet and neonatal disorders have been discussed in the context of neonatal gut microbiome. On the translational front, in-depth presentations have been made by authors in favor of the potential use of probiotics to reduce neonatal morbidity. Controversies against such use have been argued via citation of knowledge gaps and presentation of possible adverse effects. Other highly relevant topics have been covered including significant rationale and feasibility of various strategies that may restore and build healthy newborn gut microbial communities. However, these discussions and over a thousand citations have been largely limited to studies and experiences in Western countries. This is due to a serious lack of reports and experience in the Global South that can serve as a rich source of information due to its diversity and extremely large population size. In this chapter, we will introduce the magnitude of neonatal health issues and present some evidence on how one-size-fits-all may not work globally when serious neonatal morbidities are in question. Using adult literature, we will help the reader speculate the differences among gut microbiota one might encounter when examining babies from different parts of the world. We will describe our >20 years of experience that spanned from research in our laboratory and neonatal intensive care unit (NICU) in the U.S. to clinical trials in the hospital and community settings in India. It is expected that the readers will be excited to expand their microbiome search to various parts of the interconnected and dynamic world to gain insights into common as well as diverse and unique traits in the neonatal gut that dictate health or disease.

## Child health as a priority at the United Nations (UN)

Historically, health and child health have been an integral part of the UN advocacy and action items among other important issues and agendas. Globally, significant progress was made in bringing child mortality from 12.7 million in 1990 to 6.3 million in 2013. However, this fell quite short of the Millennium Development Goal (MDG) of achieving a rate of about 4 million by 2015 (a two-thirds reduction). The spirit of the fourth MDG goal of reducing child mortality was again reiterated in the third Sustainable Development Goal (SDG) designed by the UN. This was conceived as a strategy for shaping the 21st Century and superior to MDGs with more equitable aims for the global south as well as the north. This SGD goal emphasized health and well-being for all and established a target to reduce neonatal mortality to below 12/1,000 and under-5 mortality to below 25/1,000 by 2030. Unfortunately, according to recent statistics, five million under-5 children died in 2021 of which 47% were in their first 28 days of life. This shows stagnation in neonatal mortality reduction, and it is projected that 54 countries will fall short of attaining the SDG target for under-5 mortality and 63 countries for neonatal mortality. This will be due to infectious diseases, including acute respiratory tract infections, along with pre-term birth complications, and birth asphyxia, which remain the leading causes of death for children under-5.^1^

## Neonatal morbidities and mortality around the globe

Infant mortality rate (IMR), the number of deaths per 1,000 live births under 1 year of age, is one of the most sensitive and specific indices of the health status of any society or country. IMR not only provides insights into the health of the infant and mother but also the overall healthcare delivery and health status of a society. Neonatal deaths contribute to about two-thirds of infant mortality in almost all countries (developed and developing). Every year, 2.7 million neonates die around the world, of which >0.6 M die due to infection.^2^ Close to 98% of these neonatal deaths take place in low- and middle-income countries (LMICs).^3,4^ These deaths stem from 6.9 M new cases of infections every year in moderate to late preterm neonates in sub-Saharan Africa, south Asia, and Latin America and account for a loss of disability-adjusted life years (DALYs) similar to that of HIV/AIDS.^5^ These morbidities and mortalities are recorded despite the use of antibiotics, in some countries rampant and indiscriminate use of injectable antibiotics. On the other hand, there have been consistent improvements in the healthcare delivery systems resulting in a significant reduction of child (<5 yrs of age) and infant mortality in these LMICs.^6^ From a slightly different perspective, it is important to note that while the rate of birth asphyxia, another contributor to neonatal deaths has decreased due to improvements in birthing places staffed by skilled personnel, it has not affected neonatal mortality. Primary causes of neonatal morbidity and mortality in these LMICs continue to be driven by neonatal infections and preterm births.^7,8^ In this context, again, it should be recognized that preterm birth does not lead to birth asphyxia, rather an opposite correlation exists. Birth asphyxia is rare in preterm and low birth weight (LBW) infants and the rate is much higher in bigger babies. Preterm birth (~10% of all births) has plagued the entire world including developed and developing nations over many decades. There has been no reduction, but rather an increase in such birth rates, with > 90% of these births taking place in LMICs3. Late-onset neonatal sepsis, necrotizing enterocolitis, and feeding intolerance constitute the bulk of morbidities in preterm neonates pointing directly toward the need for interventions that can directly have an impact on these conditions.

## For how long can the world wait?

Careful analysis of the literature coming from the developing world setting and some groups in the West reveals review articles, meta-analyses, opinion pieces, and slogans – many in high-impact journals. However, the high rates of neonatal deaths and dismal improvement have continued for >20 years with minimal or no attempt to conduct primary studies of new interventions with some potential for success. Reports close to a decade ago suggested a possible 70% reduction in neonatal deaths by scaling up existing interventions and improving health systems.^9^ Recommendations (by international organizations such as the WHO) based on these and other primary studies conducted in high-income settings^10,11^ have not helped much in improving indices around neonatal health in 10 years. To the contrary, results of a handful of rigorously designed studies in LMIC countries have now conclusively demonstrated that such extrapolation of results can result in seriously flawed conclusions as demonstrated in the Antenatal Corticosteroids Trial (ACT) for preventing preterm labor.^12,13^ In the primary multi-country (Argentina, Guatemala, India, Kenya, Pakistan, and Zambia) report, 28-day mortality was increased significantly (RR 1.12, CI 1.02–1.22, *p* = 0.0127), so were maternal infections in the ACT arm (3% in treatment group and 2% in the controls, *p* < 0.0001).^12^ In a secondary analysis, the authors confirmed corticosteroids as the primary contributor to the effects and described PSBI (possible serious bacterial infections) as the cause of increased neonatal mortality (24% vs 20%, *p* = 0.008) observed due to treatment with ACT.^13^

Other studies conducted with scientific rigor in LMIC settings have now demonstrated a complete lack of effectiveness of previously purported antibiotic interventions against neonatal infections. In a recent report, Roca and colleagues described no difference between intrapartum oral azithromycin and placebo groups in a cohort of 11,983 women in labor on primary outcomes of neonatal sepsis or mortality.^14^ In contrast to multiple previous small- and medium-sized studies, this robust study with sufficient power demonstrated a lack of effectiveness of antibiotics in the perinatal period. Also recently, the NAITRE study team reported no reduction, rather an increase in 6-month mortality in infants treated with one dose of oral azithromycin during days 8–27 of life.^15^ The serious impact of such early antibiotics on increased antimicrobial resistance,^16^ negative influence on long-term respiratory health,^17^ and overall damaging effect on the microbiome early in life^18^ should steer the policymakers away from such interventions seeking quick and easy paths.

With these not-so-bright backdrops, it is predicted that with current trends it may take sub-Saharan Africa over a century to achieve rates of newborn survival comparable to that in the U.S.^19^ These estimates and comments do not even take into account the future of these infants (if they survive) that are prone to enteric enteropathy, gut dysfunction early in life, and stunting. How will the economy of these countries look like when one-fifth of the children today are stunted and have possibly fallen prey to this condition because of aberrant gut microbiota in the early months of life^20^?

In a recent Diplomatic Collaboration, systematic review using data from 98 studies in LMICs Wastanedge and colleagues offer some hope.^21^ After meticulous comparative analysis, the authors identified (i) thermal regulation and (ii) feeding support including probiotics to improve survival in the preterm neonates. At the same time, Kangaroo Mother Care (KMC) was also found to impart a reduction in all-cause neonatal mortality by 43% (OR = 0.57, 95% CI = 0.37–0.89), highlighting the significant potential impact of this low-cost intervention. There was a significant reduction in all-cause mortality (RR = 0.73, 95% CI = 0.59–0.90), and NEC (RR = 0.46, 95% CI = 0.34–0.61) and incidence of late-onset neonatal sepsis (RR = 0.80, 95% CI = 0.71–0.91) when probiotics were used. At the same time, several previously purported interventions that appeared promising did not meet muster when larger trials were done. The authors described three large-scale RCTs and one meta-analysis in preterm infants describing no benefit of vitamin A supplementation in reducing neonatal mortality. What was more important in the authors’ observation was that neonatal resuscitation, an established and standard practice in every delivery room in the U.S. did not have an impact in LMIC settings, demonstrating the need for post-resuscitation care.

If the scientific community and policymakers are serious about improving neonatal health without inflicting other short- and long-term collateral damage to the baby’s life, it is high time they give urgent attention to developing new, affordable, and sustainable interventions for preventing or reducing neonatal sepsis, necrotizing enterocolitis (and preterm births). Understanding the role of microbiota during the first weeks of life and utilization of intervention modalities to manipulate gut microbiota for improved structure-function of the intestine holds a strong promise against many of these high-burden disease conditions and in improving global neonatal health. To do this effectively, it is required to examine the neonatal gut microbiota in different parts of the world, understand their drivers and modulators, and design specific interventions for that setting.

## “Gut microbiota”: a vital organ, yet not the same in all

Babies acquire microbiota from their mother and the “environment” that can vary significantly depending on where they are born. Also important is their diet – whether they are breast milk or formula fed, where breast milk serves as a seeding medium with live bacteria and other factors including non-absorbable sugars (oligosaccharides) that promote the growth and proliferation of anaerobic bacteria in the distal gut. These initial traits not only play pivotal roles in major morbidities such as neonatal sepsis and necrotizing enterocolitis but also contribute immensely to maintaining homeostasis and a healthy state during the first 1–2 years of life. This is achieved via resistance against pathogens, local and systemic immunomodulation, maintenance of intact intestinal permeability, and blocking inflammation among other mechanisms. Together, these have profound implications in nutrition, vaccine response, growth, and cognition of the infant, some of which are irreversible and have life-long ramifications.^22,23^

Multiple chapters in this special issue have elegantly described the neonatal gut microbiota, its acquisition and impact on various physiologic and immune development, and its role in preventing neonatal sepsis and necrotizing enterocolitis. It is obvious from these in-depth analyses that the bulk of the inference has been accumulated from studies conducted in North America, Europe, and Australia. A small number of studies conducted in the developing economies add to this evidence. While highly encouraging, these and other studies also raise the question of universal adaptation and effectiveness. Without a clear understanding of the initial colonization patterns, and longitudinal assessment of new acquisitions, it is difficult to speculate temporal changes in different global settings that may be driving health and disease.

In a study by Chandel et al. in breast milk-fed Indian young infants using 16S rRNA gene sequencing babies harbored an extremely diverse gut flora during the first 2 months of life. Four major phyla (Firmicutes, Bacteroidetes, Proteobacteria, and Actinobacteria) were identified where Proteobacteria (64%) and Firmicutes (28%) predominated on day 7 of life. However, by day 60, there was a significant reduction in Proteobacteria (from 64% to 13%) with a concomitant increase in the other three phyla. At the family level, as expected, an abundance of Enterobacteriaceae was 64% on day 7 reducing to 12% by day 60. Enterococcaceae also reduced from 10% to 1.74% and Staphylococcaceae from 5% to 0.05% with an increase of Bifidobacteriaceae from 0.004% to 0.305%.^24^ Palmer et al. observed diversity among U.S.-born babies during early infancy similar to that in Indian babies. There was major diversity in the individual characteristics among these infants. In this study, compared to Indian infants, while Firmicutes were of similar abundance (32%), Bacteriodetes and Proteobacteriaceae were much lower, 20% and 46%, respectively. Actinobacteria were very low in abundance in American babies (1.28%) compared to 9.51% in Indian babies. The authors of this study reported the convergence of neonatal toward a more adult-like flora by 1 year of age.^25^

In a Chinese study, the fecal microbiota of healthy infants was compared with those from other countries where the authors characterized them into P, A, and F enterotypes with abundance in Proteobacteria, Actinobacteria, and Firmicutes, respectively. There was a high level of diversity at 2 months of age compared to that in the neonatal period evidenced by the increase in Veillonella, Clostridium, Bacteroides, Lactobacillus, Collinsella, and Prevotella, and reduction of Escherichia and Enterococcus. Cesarean-delivered infants in this study had enrichment of Prevotella, Streptococcus, and Trabulsiella in contrast to the enrichment of Bacteroides, Parabacteroides, and Megamonas in vaginally delivered infants. Overall, the authors identified the Chinese pattern to be of P-type (dominated by Proteobacteria) and geographic variations in these enterotypes compositions.^26^

During the last 15 years, data from adult samples have also provided some fundamental insights into the human gut microbiota. In 2007, examining the gut microbiota of individuals from the U.S., Europe, and Japan, Arumugam, and colleagues identified three distinct clusters and referred to them as enterotypes.^27^ These three human enterotypes were not nation- or continent-specific and were predominated by Bacteroides, Prevotella, and Ruminococcus.

This study showed the presence of a small and limited number of phyla which appeared to be driven by diet and drug intake. The authors of this study described that the enterotypes are driven by molecular functions, but not necessarily by abundant species, pointing toward the importance of functional analysis to comprehend microbial communities. In a Korean study, authors and described Enterobacteriaceae as the predominant subtype apart from Bacteroides and Prevotella in the Asian population.^28^ Attempting to identify signature patterns of the enterotypes in Muslim and Han Chinese individuals, Li et al. examined fecal microbiota from 48 Han Chinese, 48 Kazaks, and 96 Uyghurs. The gut microbiota of 192 subjects in this study were classified into only two enterotypes; Bacteroides and Prevotella with an association of functional characteristics of genes with genetic backgrounds and diet.^29^ These and other studies provide ample evidence that while many commonalities exist, there remain major differences in the gut microbiota of humans from different parts of the world. Addressing the possible impact of altitude, geography, and lifestyles, Lan and colleagues examined gut flora in six different geographic locations at altitudes ranging from 2800 m to 4500 m above sea level across the Tibetan plateau.^30^ At the phylum level, the adult gut comprised of Bacteroidetes (60%), Firmicutes (29%), Proteobacteria (5%), and Actinobacteria (~4%) and marked by a low Firmicutes to Bacteroidetes ratio (0.48). At an operational taxonomic unit level revealed that core microbiotas included Prevotella, Faecalibacterium, and Blautia, whereas Prevotella predominated in five of six locations. The authors’ conclusions included significant variations with increasing altitude, BMI, and age, and richness of facultative anaerobes in Tibetan guts. Gut microbiota may play important roles in regulating high-altitude and geographical adaptations. Multiple other studies from different parts of the world reveal similar information with many commonalities, but also identification of unique phyla and family of organisms pointing toward environmental exposure and diet.^31^

## Cause, consequence, or a function of age, genetic background, and nutritional needs?

Some of the above studies and other similar reports demonstrate diversity in the gut microbiota across the lifespan and point toward genetic and metabolic diversity.^32^ Examining gut microbiota from healthy children and adults from the urban U.S., rural Malawi, and Amerindians of Venezuela, Yatsunenko and colleagues showed functional maturation of the gut microbiota in the first 3 years of life in all three populations. Significant differences in bacterial gene repertoires among the three populations were noted in this study and the Bifidobacterium genus declined over age in all three populations. While this was not unexpected, the genus Provetella stood out as a discriminatory taxon in non-U.S. populations. Similar results were reported in another study where Provetella dominance was recorded in children in Burkina Faso (West Africa) compared to those in Italy.^33^ Using fecal samples from mono- and di-zygotic twin pairs, Turnbaugh and colleagues showed that the gut microbiome was shared among family members, while each individual’s microbial community varied in the specific bacterial lineages. There was a similar degree of co-variation between adult monozygotic and dizygotic twin pairs and many shared microbial genes among sampled individuals.^34^ The authors still noted a core microbiome at the functional level and attributed changes in the host physiology when this core microbiome was altered. When it comes to the fundamental functions of the microbiota, it is not surprising that there are differences in the gut microbiota in infants, children, and adults as they grow. The microbiota of babies is enriched in genes involved in the *de novo* biosynthesis of folate, in contrast to genes that metabolize dietary folate and enriched in genes encoding enzymes involved in the biosynthesis of cobalamin (B-12) in adults or with increasing age. However, differences are seen driven by geography and adult diet (more carbohydrates from Maize and cassava in Amerindians and West African populations vs. more protein and processed carbohydrates in the U.S.). Whether these changes are imparted to the growing infant as s/he weans from breast milk and is introduced to solids via transmission from mothers and caregivers whose guts have been adapted and bacterial genera selected over generations or due to pressure for survival and acquisition as a necessity in that part of the society/geography is not known. Suffice it to say that while some universal physiological changes will take place in all babies and infants, other unique changes will be incorporated based on what the infant consumes to thrive. Hence, starting with the initial weeks of life there will be a significant impact of breast milk type in breast-fed infants and more environmental impact in those receiving formula.

## Diet, environment, and exposures impacting the gut microbiota during early infancy

In the lifespan of a human being the gut microbiome undergoes subtle as well as drastic changes that are not the focus of the current chapter except challenging the readers to fathom the outcome of changes in an infant’s gut microbiota as a result of the host of exposures an infant experiences early in life. In other chapters under diet and microbiome in this special issue, the authors have already given an exhaustive description and analysis of microbiota in breast milk and the role of breast milk oligosaccharides in shaping the ultimate gut microbiota during early infancy. Older culture-based studies^35^ and culture-independent^36^ studies have reported on the diversity of microbiota in breast milk and describe their origin, temporal changes, stability,^37,38^ and their potential role in health and disease.^23^ Studies on various genera and species spanning from the well-studied breast milk Bifidobacteria on infant gut development and maturation^39^ to multiple strains of lactobacilli^36^ including *Lactobacillus salivarius* isolated from breast milk as well as infant feces^40^ point toward the possible role of probiotics in manipulating the gut microbiota.

Currently, a large body of literature exists describing how contaminants in food and air including drugs (antibiotics in particular), preservatives, artificial sweeteners, flavors, toxins, metals, plasticizers, and particulate matter in indoor and outdoor pollution affect the gut milieu that harbors bacteria, archaea, fungi, and viruses.^41^ These pollutants have been shown to change the gut microbiota and induce dysbiosis.^42^ Multimodal interactions of these pollutants with gut microbiota have been extensively studied and reviewed.^43^ Impacts of such exposures can vary depending on the types, amount, and length of exposures and the complexity can be easily imagined when the host is an adult or child with many years of exposure. However, on the surface, this may appear less severe and have limited consequences if the host is a neonate or young infant confined to the home environment created by the mother and immediate caregivers in the household where the diet consists primarily of breast milk or formula. But, in reality, these impacts are more severe and more damaging when the microorganisms get their first foothold in the intestinal lumen and struggle to establish and expand. Any damage brings deeper impacts and serious ramifications during this vulnerable period.

During the last 10 years, high levels of organic pollutants, fluorinated compounds, digoxin, polycarbonated biphenyls (PCBs), organophosphate/organochlorine compounds and their residues, hexachlorocyclohexane, microplastics, and other toxins have been reported in the breast milk of mothers in countries spanning from Mexico,^44^ India,^45^ South Korea,^46^ China,^47^ Iran,^48^ Russia,^49^ and the U.S.^50^ Some of these compounds have been found in the colostrum of mothers in rural India.^51^ Similar compounds have been identified in breast milk and dairy milk in multiple Asian countries and the United States.^50^ More disturbing than this is the relatively new information about particulate matter in breast milk. In a recent systematic review, Van Pee and colleagues have described the link between simple ambient air pollution and changes in gut microbiota demonstrated in epidemiological, *in vitro*, and *in vivo* studies.^52^ Studies from China, India, other South Asian countries, and the U.S. have shown how particulate matter (PM 2.5 and PM 10) in the air gets access to the gastrointestinal tract via mucociliary clearance and their impact on the intestinal microbiome and lipidome.^53^ Very recent reports now point toward the impact of such air pollution on human milk oligosaccharides which could have a downstream impact on most health-promoting microaerophilic and anaerobic species that consume such sugars in the colon for their survival and growth.^54^

## Environmental impacts in the global South and the Western world

It is more than evident that environmental exposures whether via food, water, or air are currently ubiquitous around the world. No country can escape from their baneful impact. When we consider the developing country settings, it is not difficult to appreciate how population explosion, sub-optimal sanitary environment, unregulated industrial growth, and lack of or poor compliance with environmental regulations have made the scenario worse. It is also very likely that these types of environments are going to get worse where millions of young infants will grow into adulthood. Hence, it is all the more important to fully understand the biological basis of such exposures in these resource-limited settings and develop mechanisms to mitigate damage and restore healthy microbiota, which in turn, will have a long-lasting impact on the health of the citizens.

## Manipulation of the neonatal gut microbiota in a global setting

This section is a logical next step in putting gut microbiota into action while keeping the available information described in previous paragraphs in perspective. It is interesting to note that the scientific community has just begun to seriously ask the question “Why – for what”? To prevent a disease, treat a condition? Or simply for healthy growth of an infant in New York City, the Gambia, in coastal villages of India, or breast milk-fed babies of tribes that lived on millets (and all natural food) until several years back when free rice or wheat was introduced to them as a yardstick of development? The answers are quite different to each of these questions, and we do not have concrete answers for any at this point.

However, based on available data from Western countries and some from developing country settings including our own, it is not difficult to have the primary endpoint focus on the prevention of neonatal sepsis, the biggest killer of neonates using probiotics, prebiotics, and synbiotics. Manipulation of the gut microbiota to affect sepsis may have other concomitant benefits as well. In the following section, we will describe our approach and experience in conducting NIH-funded laboratory, hospital-, and community-based research that spanned >20 years to culminate in a large randomized clinical trial (*n* = 4556) of synbiotics in India which showed a highly significant reduction of sepsis and death (NNT = 27) in the treatment arm. This study was terminated early by the DSMB due to a demonstration of effectiveness.^55^ Before we do so, we will present the results of basic and clinical research conducted in our laboratory that provided the rationale and required level of comfort and confidence to launch a trial of this magnitude.

## Pathogenesis of disease and strain selection

Even if we have forgotten about the first controlled Scurvy trial of James Lind in 1747 or the randomized control trial of streptomycin in pulmonary tuberculosis 200 years later in 1946 by MRC of the UK^56,57^ in the current day of medical research we are all familiar with Phase I (safety/toxicity) requirements and Phase II and III efficacy trials before any new drug/molecule is ready for FDA approval. We are also aware that any trial is preceded by many years of bench research, and undergoes various modeling including *in vitro* tissue culture, followed by animal models that mimic (to the extent possible) human disease. The researcher always starts with the pathogenesis and mechanism of the disease in mind and attempts to either cure or prevent it by breaking or modifying the steps that are known to produce the disease state. It is quite surprising and disappointing to note that almost no human probiotic trial, especially those in necrotizing enterocolitis or sepsis started with these steps. Investigators around the Western world used one strain or the other whichever one was available to them. Also, until recently, most of the investigators did not bother to examine colonization of the strain during or after therapy. Possible impacts of geographic difference, the mother’s secretor status, or other factors that could change the breast milk, and in turn, the developing gut flora in the virgin intestine of the newborn were never considered. Different strains or strain combinations were used by each group and with a few exceptions, the same preparation was never used by other investigators for the same conditions under the same protocol. How can we then expect to have a unified answer? Meta analysis are helpful, but what good are those if they end with a statement that “probiotics should be used in preterm infants as long as an appropriate strain is available for the population in which it is intended to be used and the correct dose is known”?

## Research on preterm gut microbial ecology and NEC in the early 1990s

Examining the pathogenesis of NEC and looking for an infectious etiology, Gupta et al. in our group published the first bold and negative report demonstrating a clear “lack” of association of any previously purported infectious agent in preterm babies with NEC compared with controls.^58^ In a comprehensive analysis of bacterial, parasitic, and viral agents in stool samples of 23 NEC, 23 matched, and 10 random controls, Gupta showed that NEC babies were not associated with any virulent/pathogenic bacteria, nor did the *E. coli* and Klebsiella strains had increased beta-galactosidase or delta-hemolysin activity as previously purported. Astrovirus was identified in a stool sample from one control infant. *E. coli* strains from NEC babies although highly adherent to Caco-2 cells did not belong to entero-aggregative, entero-invasive, or entero-pathogenic groups. An extremely important and relevant observation in this study was that eight infants were bacteremic and in 7 of 8 the same organism was present in the stool as demonstrated by molecular typing.

Building on these findings, our group further observed adherence of *E. coli* strains and colonization pattern (Gram-negative and Gram-positive combination in tissue culture models) to be important in NEC pathogenesis. After unsuccessful attempts in rats, mice, rabbits, and newborn mice, we could produce NEC-like disease in ileal loops of weanling rabbit pups using the adherent *E.* *coli* and Klebsiella strains and block the same by co-infection with Gram positives from control infants such as *Enterococcus faecium*.^59^ In the weanling rabbit ileum, we found clumps of *E.* *coli* in the submucosa with accompanying inflammation that had spread to deeper layers. These phenomena were drastically reduced and except mild submucosal edema, no injury was seen in loops co-infected with *E. faecium*. The next logical step in our investigations was to further examine the bacterial translocation, something we did not expect to happen when normal flora *E. Coli* was used and in a tissue culture transcytosis model observed significant translocation of *E. Coil* to the lower chamber and blockade of the same when Gram-positive normal flora enterococci and staphylococci were used.^60^

While this was not exactly an Eureka moment for our group, we could easily hypothesize that the “ecology” and “absence” of good Gram-positive organisms were instrumental in NEC pathogenesis and the ability of Gram negative bcteria to adhere to and cross epithelial cell layer s a critical initial step in triggering inflammation and a cascade of events which ultimately lead to NEC. At the same time, our enthusiasm was dampened by our recognition that avirulent Gram positives that were protective in our models including coagulase-negative Staphylococci are responsible for a significant portion of late-onset sepsis in preterm babies, and hence, could not be used to manipulate the infant gut flora.

## Translational endeavors by our group

Knowing fully well that we could not use these Gram-positive organisms in our babies, we quickly looked up to probiotic strains and new funding from Fogarty International Center (NIH) allowed our colleagues in India to study the ability of LGG (*Lactobacillus rhamnosus*) to colonize the neonatal gut. LGG at that time had the largest literature backing its health-promoting effects on multiple conditions. In a prospective study, we randomized 71 preterm infants weighing <2000 g at birth. Infants <1500 g birth weight (24 treated, 15 control) received 1 billion colony count of LGG orally twice daily for 21 days and those weighing 1500–1999 g (23 treated, 9 control) were treated for 8 days. Stools were collected before treatment and on days 7 to 8 (and days 14 and 21, in infants weighing less than 1500 g) for quantitative aerobic and anaerobic cultures. Colonization with LGG occurred only in 21% of infants <1500 g and 47% in larger infants and was limited to infants who were not on antibiotics within 7 days of treatment with LGG.^61^ With such results on colonization, we had no option other than searching for a strain that could do a much more efficient job of colonizing in a controlled hospital environment so that when we move to a community setting with many exposures and overriding variables, we would still expect robust colonization to yield the effectiveness on blockage of adherence and translocation by Gram-negatives.

## Search and identification of an appropriate strain

Concurrently our laboratory was engaged in examining multiple probiotics strains from USA, India, and Tatarstan (former Soviet Union) brought to us by our colleagues and also included our new library of lactobacillus and bifidobacteria strains that could be cultured (under microaerophilic and anaerobic environment) from stool samples (diapers) collected in our NICU, well-baby clinic and pediatric practice. Of a total of > 280 strains (including strains marketed as a supplement), we observed the desired traits (highly adherent to cultured Caco-2 cells, ability to block adherence and translocation of E. coli, and block NEC-like injury and sepsis due to bacterial translocation in the animal model) in only one *Lactobacillus plantarum* (now called *Lactiplantibacillus plantarum*) strain ATCC-202195. None of the > 13 bifidobacteria, > 15 *L. acidophilus*, or six other *L.* *plantarum* strains in our collection exhibited even a low level of effect in our models. A handful of strains belonging to other genera that showed some degree of protective potential were excluded from further investigations due to the distinctly superior effect of strain ATCC-202195.

## Preparatory hospital-based study in Indian neonates to examine colonization

Our previous experience with poor colonization with LGG, prompted us to do a hospital-based study in India before embarking on the large efficacy trial. In this RCT, we enrolled 31 inborn newborns >35 weeks of gestational age and >1800 g birth weight and randomized them to receive the synbiotic (*L. plantarum* + FOS) or dextrose saline placebo once daily in a 2:1 allocation for 7 days. *L plantarum* was cultured from the stools of 84% of the treated infants after 3 days of treatment, and from 95% of infants on day 28 after birth. Of the infants, 100%, 94%, 88%, 56%, and 32% remained colonized at months 2, 3, 4, 5, and 6, respectively.^62^

## Preparations and launching of a well-powered RCT using the L. plantarum in Indian neonates

Our continued NIH U0–1 funded rigorous work on population-based surveillance of neonatal sepsis at two large field sites in India (that included coastal, tribal, and peri-urban slums) had started revealing by this time that majority of neonatal sepsis in India was of late-onset. We also observed the GI tract as the source of a significant percentage of organisms cultured from blood, similar to what we had seen in our very first studies in the U.S. NICU. With evidence from the laboratory studies and preliminary clinical experience on *L. plantarum* as a potential candidate, our proposal to NIH to use *L. plantarum* was considered highly meritorious by the study section. At the same time, we also confronted red flags with the valid concerns that no animal toxicity studies were done on this strain. Accepting this requirement, we pointed out that there may not be any benefit of doing traditional rodent toxicology studies because this strain or any other enteric Gram-negative strains in our collection produced any disease or injury in rats, mice, or even neonatal mice. Hence, traditional toxicology studies done for evaluating pharmaceutical products may have minimal or no utility while examining this biologic. A chronic toxicity study was then launched in newborn rabbits (disease model for sepsis and NEC) at an AAALAC-accredited facility to conduct the studies under GLP guidelines. Daily oral feeding was done for 28 days followed by exhaustive pathology and clinical chemistry. The 4 weeks of oral administration resulted in normal growth of the rabbit pus and the intervention was declared as safe with no unexpected adverse reactions.

## Launching of the field RCT in the Indian community setting

As our group began preparation for the community trial, the new R0–1 funding was received and the fully mature research sites developed over a 10 yr period, highly competent local PIs, and fully trained hospital, lab, and community personnel (total >250 and one in each village and supervisors at two levels) were available to undertake implementation of the clinical trial protocol. Since this was different from surveillance and much larger in scope, the research team had to patiently appreciate the need for scores of approvals. This included review and approval by the Scientific Review Committee of the Indian Council of Medical Research (ICMR), Ethical Committee approval by ICMR, and finally a review from security and other ancillary angles by the HMSC (Health Ministry Screening Committee of the Government of India). The assistance and patronage received from the senior leadership at the Federal level including those from the Director General of ICMR was unparalleled. However, due to the frequency at which these committees met and then another series of approvals from similar State Government committees in Odisha (where the study was undertaken) took the bulk of the time before the local hospitals did their own review and hospital IRB approval. These were all done after the study was funded by the NIH and all other U.S. institutional approvals (including IRB) were in place. From a U.S. standpoint, Indian IRBs had to be revamped to meet OHRP (U.S. Dept of Health and Human Services) requirements and registered with the U.S. agency. Lastly, the FWA (Federal Wide Assurance) registration had to be established for U.S. federal fund transfers to India.

Finally, 4,556 infants were enrolled that were > 2,000 g at birth, and >35 weeks of gestation, had no signs of sepsis or other morbidity, and were monitored for 60 days. There was a significant reduction in the primary outcome (combination of sepsis and death) in the treatment arm (risk ratio 0.60, 95% confidence interval 0.48–0.74). Significant reductions were also observed for culture-positive and culture-negative sepsis and lower respiratory tract infections. Following WHO guidelines for neonatal sepsis called PSBI (possible severe bacterial infection), lower respiratory tract infections were recorded separately and also had a significant reduction pointing toward immunomodulation by either the *L. plantarum* strain alone or the modified gut microbiota discussed in detail in the original article.^55^

## Lessons learned from the global endeavor

**Figure 1.**
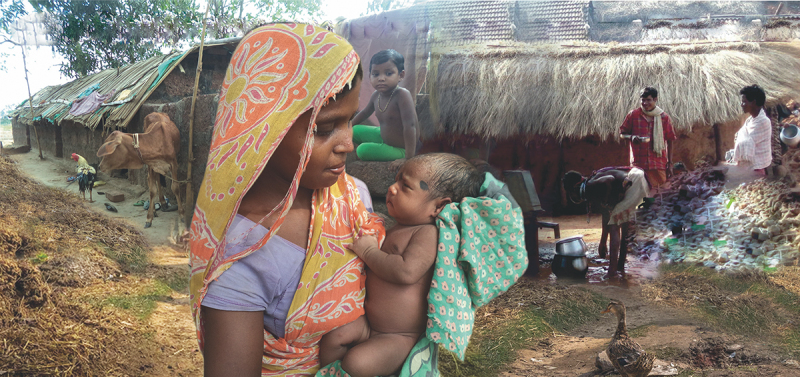
A typical Indian village; home to the bulk of deaths due to neonatal sepsis.

Despite urbanization and industrial growth, the majority of Indians live in rural areas (Figure 1), and so are the citizens of most other countries in South Asia and sub-Saharan Africa. These settings are also home to the one-fifth of all children who are stunted. While remarkable improvements have taken place in India under the Swachh Bharat Mission (a sanitation campaign by the Prime Minister) and improvements in the healthcare delivery systems, the bulk of rural Indian babies grow up with animals, birds, and suboptimal sanitation. These exposures may allow more “diversity”, considered better than infants growing up in cleaner westernized societies who eat more processed food high in fat and receive many doses of antibiotics by the time they are in kindergarten. Such settings (including those in European rural/farm areas) are known to provide protection against allergy and atopy driven by a quick shift from Th-2 to Th-1 type immune response. In developing country settings, in addition to this, infants also receive an early and heavy dose of microbial exposure, many with known virulence traits and enteric disease potential. They get breast milk fed by their mothers who have experienced lifelong WASH (water, sanitation, and hygiene) issues and are now victims of new mining, industrial exposures, and vehicular emissions along with the severe impact of global warming and climate change. Hence, it is not prudent to expect their gut flora to be developing at the same pace, manner, and diversity as in Western countries. The population density itself makes South Asian countries like India, Pakistan, and Bangladesh more challenging than countries in sub-Saharan Africa. These, when coupled with a diverse diet – some completely vegetarian, others eating but unable to afford animal products, rice vs. wheat, vs millet eaters in different parts create an unfathomable array of diversity all affecting the gut microbiota of pregnant women, lactating mothers, and young infants.

It needs collaborative efforts by country leaders, global health agencies, and scientists to identify and freely and quickly disseminate the lessons learned in each setting to design interventions for the young. Sometimes it may be a boutique therapy only applicable to one society, but in many others, generic lessons can be broadly translated into practice. We can ensure healthy infancy through resilient adulthood if this is taken up as an ongoing task with an open mind to change as and when required.

## Role of prebiotics in the neonatal synbiotic preparation

After unsuccessful attempts in making Lactobacillus GG colonize the neonatal gut of our colleagues in India and then similar negative results with *L. sporog*enes at the same center (unpublished data), we wanted to put all our efforts for the best outcome and launched the first synbiotic study in neonates with FOS. Among a few oligosaccharides available in the market, nutraflora Fructo-oligosaccharides, FOS had the U.S. FDA GRAS status, and the high-quality bulk product was available readily. We did not have the opportunity to examine the *L. plantarum* strain without FOS, nor did we use any other prebiotic. The role of other non-absorbable oligosaccharides and human milk oligosaccharides need to be assessed in combination with candidate probiotic strains for evaluation of the efficacy of synbiotic products.

## Return on investment

When one says “global”, the West is not isolated and the benefit derived from such research immediately spills over to all. The innovation made in one place is shared by the world community. What took us 20 years to accomplish can be done in one-tenth of the time if we were to launch the same research endeavor now. Imagine enrolling 4,500 babies in a Western setting. How many centers will it take and at what cost to complete the trial in 3 years? We don’t need to do culture-based analyses of microbiota today, data collection on paper forms and double entry into the computer have been history even in the same setting for more than 10 years. The younger generation is more adaptive to collecting data on tablets and cell phones. Most people, even in remote rural areas have a cell phone and internet connectivity (even if there is no potable drinking water). Rudimentary government systems have been replaced with digital surveillance systems. If multiple investigators with different interests and expertise sit together to plan research studies, at least 10 studies can be conducted concurrently using the same platform and research staff. While we did not have time or energy to look beyond babies, if organized from the beginning, the population of the same communities can be utilized not just for research in neonates, but in mothers, adolescents, adults, and the elderly. Longitudinal assessments in such cohorts are gold mines for innovation, discovery, and development.

## Practical and logistics challenges for interventions using live bacteria

We have not described the uphill battle we had to face to keep probiotic bacteria alive. Under a research environment, we stored clinical trial material at − 80^0C^ at our research hospitals and distributed monthly to district hubs where those were stored under refrigeration (for a month). The preparation was left in the baby’s home for a week where temperature could reach 110–150 ^0F^ during summer months. We had to conduct an intensive stability testing (that continued till the end of the study) of our preparation in the lab making sure that there was no drop in viability for over 2 weeks at such extreme temperatures. How can the lyophilized powder be reconstituted where drinking water could be highly contaminated and be a source of infection for the baby? Again, in a research setting, single-use sterile dextrose saline vials, and sterile plastic containers for reconstitution and syringes were used for daily feeding by the trained healthcare worker. Can the mother feed their babies without any outside help?

Those conducting implementation research and scaleup may argue, citing the difficulties even with vaccination during infancy and childhood. In our study, there was a need for a 7-day dosing to be protective over 2 months. Researchers and commercial companies are now working on different lyophilization processes and adding stabilizing ingredients to keep live bacteria active at higher temperatures. Probiotics are available in many different forms other than powder/capsules (drops, oil/emulsion suspensions) for ease of administration. Many in this field think that spore-forming strains may be the best practical answer that will not need lyophilization (as long as candidate bacterial strains are available). Scientists are now engaged in making the preparations sturdier and more acid and alkali-stable by various coating methods including some with specific nano-particles. Can there be a single-dose probiotic that can be administered at birth to stay colonized for an extended period and provide protection? Can administration of the probiotic to the mother be a better solution? Studies are ongoing at the Kintampo Health Research Center in Ghana where pregnant women are fed probiotics in their third trimester with an expectation that their gut will be colonized and transmit the organisms to the baby during vaginal delivery and in the first weeks of life when they are most vulnerable to infections. Examination of breast milk in these studies will also shed light on the transport of specific probiotic bacteria from the mother’s gut to her breast milk which can be an easy and safe mode of delivering specific probiotic strains to the neonate. Rocket science is not required to answer these questions. Persistent efforts and commitment to improve neonatal and child health, not by a handful of experts, but ownership and involvement by all stakeholders will ensure that all who need microbiome-based therapies can get it.

## Quality control, contamination, and adverse events

Probiotics have been used in yogurt and other fermented food products for many years and some strains are now considered under the GRAS (generally regarded as safe) category by the food FDA. However, when it comes to use in neonates and infants, special care needs to be exercised to handle their immature gut integrity and underdeveloped immune systems. Contamination with fungal strains during production can be problem^63,64^ and although rare, multiple adverse events have been reported in neonates and infants after receiving probiotics.^65^ These events are uncommon but attract attention due to the devastating consequences, and every measure should be taken to avoid them. Recently, Shane and Preidis provided a timely viewpoint recommending a framework for manufacturers and healthcare providers summarizing different steps to be taken and laboratory analysis limits to be followed for products to be used in the NICUs.^66^ As medical researchers, it is our job to weigh risks and benefits while conducting clinical trials and the highest level of care needs to be imparted while dealing with neonates and live microbial products. As clinicians, we have taken the Hippocratic oath to do no harm, but we also need to “exercise our greatest ability and judgment” to use dietary regimens that will “benefit” our patients. In 2019, the U.S. FDA and NIAID jointly organized a workshop to deliberate on the delicate topic of “Science and Regulation of live microbial products when used to prevent, treat, or cure diseases”.^67^ We described our clinical trial of synbiotics supplement that was not done under an IND (investigational new drug), but all the stringent and required steps were taken by us with the cognizance of the age and vulnerability of our subjects (neonates). We also participated and agreed with many others that probiotics should not be regulated as drugs for all populations for all health-promoting use. Those types of regulations will discourage further clinical development and make the cost prohibitive for general populations.

## Financial implications and economic drivers for the use of probiotics in the global south

From the experience gathered while implementing the clinical trial in India, we estimated that the cost for a one-week treatment with probiotics is ~ $1 and easily surpasses any bar set for cost-effectiveness analysis (with an NNT of 27, the cost would be $27 to prevent one case of neonatal sepsis). However, there remains the cost of storage (refrigeration needed for current preparations) and distribution. Since most countries have cold-chain arrangements for vaccine storage and delivery, this is feasible, but the bulk of probiotics may be prohibitive if existing systems need to be utilized. Alternatively, one needs to focus on reducing the cost of bulk production and accept some loss of viability without refrigeration. Most supplement companies adopt this approach now and pack more CFUs per unit to cover loss during storage. This is not always easy and is driven by the particular strain in question where the same fermentation can produce 50–500 times more bacteria compared to another strain. However, since the effectiveness of any strain is of paramount importance, it is a difficult balance to maintain. A recent trend in the probiotic supplement industry, “more” has graduated into “mega” and manufacturers are packing 10 to 100 billion CFUs of probiotics in one dose. This practice may be acceptable with adults but not for children, especially never for neonates, and more is not always better. In fact, an extremely high initial dose capable of quickly expanding several logs in the colon can create adverse impacts. Such overdosing or bombardment of the naïve gut with immature tight junctions can result in local inflammation and translocation across mucosa and sepsis by the probiotic strain itself as reported in the recent FDA warning. Also, in our opinion, there is no need to administer such a high dose of live bacteria. If the strain is a good colonizer, even a few that cross the acid and alkaline barrier will slowly proliferate in the gut. The industry will be better served by providing a protective coating to the bacteria (and administering a smaller and defined dose), but that too may not be easy and cheap while dealing with live bacterial preparations.

The above discussion is relevant only if a product is available to manufacture and be sold (not taking into account the cost of developing the same and any profit). When it comes to innovation and discovery, as it is in the case of gut microbiota manipulation, it is purely driven by the return on investment. If it is a patented drug or small molecule with the potential to treat cancer, there is no need to influence any policymaker or philanthropy. Pharmaceutical companies or the “innovation wing” of large philanthropies flock to own it and develop it further. But, when products like probiotics or prebiotics modulating gut microbiota without intellectual property protection are in question, and the biggest customer base is in the Global South, the only solutions are probably the free market and government initiatives. Economies in developing nations have fortunately improved to the extent that parents can afford a product that is available in the market at or slightly above the cost of production.

In any innovation, there is an inherent intent to invest in the product for a profit and it also comes with a risk. Affluent and resource-constrained societies and governments take different levels of risks, and some may not be able to take any risk at all. Fortunately, research and development in the field of probiotics or fermented products and supplements do not require extremely large investments. Many countries and societies already have a track record of using epidemiological evidence and experience to treat their populations and the lowest-hanging strains can be examined quickly. For example, if we consider other countries using the *L. plantarum* − 202195 strain that showed excellent traits for the prevention of neonatal infections, there will be no need for safety studies, nor will there be a need for a full-blown RCT. However, the critical element of colonization in babies and the changes it induces in the gut microbiota in the new population needs to be examined. If it colonizes well during an extended period, one could propose with confidence that similar results will be obtained in the new setting. A bridging RCT or open-label observational assessment can follow in a much smaller population costing a fraction of a typical multi-phase RCT leading to the standard of care. Not relevant for neonates, but when used for older children or adults, many sub-Saharan countries have successfully tried the model of country kitchen and making medicinal yogurt using starter cultures of selected well-researched strains, an approach that is significantly less expensive and sustainable. Such paths need to be carefully chosen and not mixed up with interventions where a defined drug-like response is expected at a defined dose for a defined subject population to treat or prevent a defined medical condition.

## Concluding remarks

Today, the lay public and many informed health providers believing in the manipulation of the gut microbiota expect it to work like a miracle. They compare probiotics to broad-spectrum antibiotics forgetting the resources that have gone into developing antimicrobials over the last 95 years. Others including major philanthropies support studies in resource-constrained countries to examine if oral antibiotics can be given to all children in the population. It is extremely discomforting to read reports in premier medical journals where antibiotics were given to newborn infants in the first month of their lives to reduce under-6 month mortality. Which lethal conditions the single dose of antibiotic will prevent for up to 6 months? What are the other consequences? Is it scientific or for that matter even ethical and responsible conduct to expose an entire healthy neonatal population to such drugs? When the livestock industry in the West is moving away from giving antibiotics in the poultry feed to cater to the discerned customers, the same informed individuals and agencies promote another group of industries overseas that has not come up with a new antimicrobial in decades. Instead of flooding the healthy human gut and already polluted environment with more antibiotics, we will serve the mankind better by understanding the underlying biological mechanisms of damage to the gut flora by pollutants in air, water, and food.^68^ Our colleagues in environmental sciences should invest in developing bioremediation bacterial strains to mediate environmental pollution,^69^ especially those with the ability to degrade antibiotics and mitigate resistance and ecotoxicity.^70^ We the medical scientists will do our part to manipulate and employ the 10^13^ microbial army in the gut to modify the ingested chemicals, heavy metals, and pharmaceutical pollutants^71^ and introduce new strains with specialized traits to transform and degrade them in the gut to improve health.^72,73^

There is a need for innovation that is feasible, affordable, culturally acceptable, and sustainable. As pediatricians, if we were to take the UN Secretary-General’s global strategy and call for developing cost-effective simple modalities to reduce mortality and morbidity in newborns in LMICs via the “Every Newborn Action Plan” seriously, it should not warrant much discussion to put microbiota on the forefront and design region-specific interventions to modify the same. This can be done at a fraction of the cost compared to other interventions to save the world’s youngest citizens from dying and may protect them from developing irreversible conditions such as stunting with many negative ramifications during adulthood.
